# Impacts of the 13-Valent Pneumococcal Conjugate Vaccine in Children

**DOI:** 10.1155/2015/591580

**Published:** 2015-08-17

**Authors:** Susanna Esposito, Nicola Principi

**Affiliations:** Pediatric Highly Intensive Care Unit, Department of Pathophysiology and Transplantation, Università degli Studi di Milano, Fondazione IRCCS Ca' Granda Ospedale Maggiore Policlinico, Via Commenda 9, 20122 Milan, Italy

## Abstract

Applications of the heptavalent pneumococcal conjugate vaccine (PCV7) in the pediatric immunization schedule have dramatically reduced the incidence of pneumococcal diseases in both vaccinated children and unvaccinated individuals of all ages. However, increased infections caused by non-PCV7 serotypes have been reported by several groups. To overcome this problem, new vaccines covering more serotypes including the emerging serotypes have been developed. The 13-valent pneumococcal conjugate vaccine (PCV13) currently covers the 7 PCV7 serotypes (4, 6B, 9V, 14, 18C, 19F, and 23F) and 6 additional serotypes 1, 3, 5, 6A, 7F, and 19A. After the first year of PCV13 applications in the immunization schedule in young children, global evaluation studies demonstrated that PCV13 provided a wider coverage and more effective prevention than PCV7 against invasive pneumococcal diseases (IPDs), mucosal pneumococcal diseases, and pneumococcal carriage. We reviewed the effects of PCV13 in the control of pneumococcal diseases in children based on previous studies.

## 1. Introduction

The heptavalent pneumococcal conjugate vaccine (PCV7) schedule in the pediatric population has significantly reduced the incidence of pneumococcal diseases in both vaccinated children and unvaccinated individuals of all ages [1]. This led to the conclusion that PCV7 not only was highly effective in vaccinated children but also could induce herd immunity, which limited the spread of pneumococcal diseases in the population living in the same geographic areas as the vaccinated children. For example, the US CDC has reported up to about 90% reduction of the incidence of invasive pneumococcal diseases (IPDs) caused by* Streptococcus pneumoniae* in young children with the introduction of PCV7 [2]. After the applications of PCV7, a significant decline in pneumococcal mucosal diseases such as acute otitis media (AOM) and nonbacteremic pneumonia has also been reported worldwide in children and in adults, especially in the elderly [3, 4]. While the incidence of pneumococcal diseases caused by PCV7 serotypes continued to decline with the introduction of PCV7, increased incidence of infections caused by non-PCV7 serotypes, mainly serotypes 19A, 7F, 6A, and 6C, has been reported by several groups [1], which reduced the global efficacy of PCV7 against pneumococcal diseases [5–7]. To overcome this problem, a new vaccine covering more serotypes, especially the emerging serotypes, has been developed. The 13-valent pneumococcal conjugate vaccine (PCV13) currently covers the most serotypes, including 7 PCV7 serotypes (4, 6B, 9V, 14, 18C, 19F, and 23F) and six additional serotypes 1, 3, 5, 6A, 7F, and 19A. The carrier protein used in PCV13 remains the same as that in PCV7. Thus, all the capsular polysaccharides of the 13 serotypes included in PCV13 were conjugated with the nontoxic mutant of diphtheria toxin CRM 197. PCV13 was licensed to replace PCV7 in 2010 for children between 6 weeks and 5 years of age in the United States of America and the European Union [8, 9]. PCV13 was licensed on the basis of immunogenicity data alone through a putative protection correlate derived from pooled immunogenicity data and results of pediatric efficacy trials on PCV7 [10]. Therefore, assessments of the protection effectiveness of PCV13 have drawn great interest immediately after it was licensed. Given the limitations of immunogenicity data in predicting protective efficacy conferred by vaccines [11], postmarket surveillance and effectiveness studies are highlighted for evaluating newly developed vaccines, particularly those with additional serotypes such as PCV13. Therefore, a number of studies have monitored the incidence of pneumococcal diseases in PCV13-vaccinated and PCV13-unvaccinated population and the circulation of pneumococcal serotypes in both patients and healthy subjects. In this review, we evaluated the impacts of PCV13 in younger children based on previous studies on the effects of PCV13 on the incidence of invasive pneumococcal diseases, pneumonia, acute otitis media (AOM), and nasopharyngeal carriage. We searched PubMed for eligible studies published from January 2010 to August 2014 using the key words, “PCV13” or “13-valent pneumococcal conjugate vaccine” and “children” or “pediatric.” Only articles published in English were included in the evaluation.

## 2. Invasive Pneumococcal Disease (IPD)

Most studies on the effectiveness of PCV13 against IPD have demonstrated that PCV13 introduction has significantly reduced the incidence of IPD in both vaccinated children and unvaccinated population compared to the previous PCV7 applications, and the results were independent of countries, the scheme of PCV13 administration [12–18]. However, different effectiveness regarding the 6 additional serotypes has been reported.

The traditional 3 + 1 scheme of administration at 2, 4, 6, and 12 months was used in the USA. Recently, the US Centers for Disease Control and Prevention (CDC) have evaluated the impacts of PCV13 on IPD incidence through an active population-based surveillance in 10 regions around the country [12]. Continuous reduction of IPD cases due to new serotypes included in PCV13 has been observed in all groups of different ages during the first three months after the introduction of PCV13 in 2010. Particularly, 93%, 75%, 72%, 62%, and 58% reductions of the incidence of IPD caused by serotypes 1, 3, 5, 7F, and 19A were observed for subjects of <5, 5–17, 18–49, 50–64, and >65 years of age, respectively, in 2012 and 2013. However, analysis of the incidence of IPD caused by non-PCV13 serotypes revealed a possible early evidence of serotype replacement among adults of 18–49 and 50–64 years. In these two groups, incidence of IPD caused by non-PCV13 serotypes in 2012-2013 was 13% and 26% that were higher than expected when PCV13 was not available. This had only a marginal impact on the global effectiveness of PCV13 because of a significant reduction (about 30,000 cases) in overall IPD incidence and mortality (3,000 deaths) in all groups of different ages. The most significant reduction of IPD incidence was observed in the group of children aged <5 years for whom the IPD incidence was reduced by 64% in 2012-2013 compared to the time when PCV13 is not available. On the contrary, reduction in mortality was more important in adults, particularly in those older than 50 years.

It has been reported that the protective effectiveness of each serotype included in PCV13 was different. For example, it was 100% for serotype 7F, 76–96% for serotype 19A, and 13–93% for serotype 3. In addition, PCV13 administration was significantly effective in a special population, the Alaska Native people who are under higher risk of IPD than the general population of the USA. Singleton et al. have followed a total of 3,714 Alaska Native children (<5 years of age) who received the PCV13 vaccination between January 2009 and August 2011 according to the US recommendations [13]. They found only 9 cases of IPD including 7 cases caused by PCV13 serotypes between 2009 and 2011 (106.7/100,000/year), whereas 52 IPD cases including 31 cases caused by PCV13 serotypes were diagnosed between 2005 and 2008 (399.0/100,000/year; *P* < 0.001). In addition, no IPD cases were reported among PCV13 recipients during 2009–2011.

The 3 + 1 schedule was also applied in Canada and led to highly protective effectiveness. After the introduction of PCV13 in Canada, the incidence of IPD in children <5 years of age declined from 18.0 to 14.2 cases per 100,000 population in the period of 2010–2012 [14]. Specifically, PCV13 serotypes declined significantly from 66% (224/339) to 41% (101/244; *P* < 0.001) in children <5 years of age and from 54% (1,262/2,360) to 43% (1,006/2,353; *P* < 0.001) in children ≥5 years of age. Serotypes 19A, 7F, 3, and 22F were the most common serotypes in IPD cases reported in 2012. The IPD caused by serotypes 19A and 7F declined from 19% to 14% (*P* < 0.001) and 7F from 14% to 12% (*P* = 0.04), respectively, during 2010–2012. IPD caused by serotypes 22F and 3 increased from 7% to 11% (*P* < 0.001) and 7% to 10% in children of <5 years of age (*P* = 0.22), respectively, during 2010–2012 [14].

The 2 + 1 schedule (PCV13 administration at 2, 4, and 12-13 months) was started in England, Wales, and Northern Ireland on April 1, 2010, to replace the previous PCV7 schedule. According to the epidemiological data in the Public Health of England, 706 IPD cases were reported during April 2010 and October 2013, including 30 caused by PCV7 serotypes, 292 caused by the additional 6 serotypes of PCV13 or serotype 6C, and 414 caused by non-PCV13 serotypes [15]. Regarding the vaccination status of the studied children, it was reported that the PCV13 effectiveness after 2 doses in the first year or one dose after 12 months was 75% (95% confidence interval [CI], 58%–84%). The effectiveness was 90% (95% CI, 34%–98%) for the PCV7 serotypes and 73% (95% CI, 55%–84%) for 4 of the 6 additional serotypes of PCV13. In addition, the vaccine effectiveness of serotype 3 was not significantly different and the vaccine effectiveness of serotype 5 could not be analyzed because no case of infection due to this serotype was observed during the studying period [15].

In Denmark, a nationwide population-based cohort study was conducted to evaluate the dynamic changes of IPD incidence during three periods, the baseline (2000–2007), the PCV7 period (2008–2010), and the PCV13 period (2011–2013) [16]. In the study, a 21% reduction (95% CI, 17%–25%) of IPD incidence in the whole population and a 71% reduction (95% CI, 62%–79%) of IPD incidence in children aged <2 years were reported after PCV13 introduction. In addition, a 28% reduction (95% CI, 18%–37%) of the 30-day mortality due to IPD was observed, decreasing from 3.4 deaths (95% CI, 3.2%–3.6%) to 2.4 (95% CI, 2.2%–2.7%) per 100,000 population after PCV13 introduction. The decline of mortality was observed in all groups of different ages and mainly in the nonvaccinated population, which is consistent with the report from the USA [16]. However, for serotypes 1 and 3, there were no significant changes in incidence beyond what would be expected from the natural cyclical patterns [16]. On the contrary, for serotype 19A for which a significant increase in disease incidence following PCV7 has been reported, the introduction of PCV13 was followed by a significant decline in the number of the cases towards baseline pre-PCV7 levels.

The high effectiveness of PCV13 (the scheme  2 + 1) was also reported in Israel by Ben-Shimol et al. based on a prospective surveillance of IPD during July 2004 and June 2013 [17]. The incidence of IPD caused by PCV7 6A serotype during the PCV13 application period decreased by 95% (incidence rate ratio [IRR] = 0.05; 95% CI, 0.03%–0.09%) including 90% in the PCV7 period and further 5% in the PCV13 period among the 2,670 IPD cases compared to the pre-PCV13 period. The incidence of IPD caused by the 5 additional PCV13 serotypes 1, 3, 5, 7F, and 19A increased initially by 47% but subsequently decreased by 79%, resulting in an overall 70% reduction during the entire study period (IRR = 0.30; 95% CI, 0.21%–0.44%). In total, a 63% reduction of IPD caused by all serotypes was observed in children aged <5 years (69% and 48% in children <2 and 2–4 years of age, resp.). However, a certain degree of replacement was evidenced because a twofold increase of IPD caused by non-PCV13 serotypes was found (IRR = 2.43; 95% CI, 1.73%–3.66%). The most commonly increased serotypes were 12F, 15 B/C, and 33F [17].

On the contrary, negative replacement phenomenon was reported in a study conducted by Levy et al., in which 1,406 cases of pneumococcal meningitis were reported in 2001–2012 [18]. Three years after the PCV13 administration, the number of pneumococcal meningitis cases significantly decreased by 27.4% (*P* = 0.041). Specifically, a 28.2% (*P* = 0.039) reduction was reported for children <2 years of age. Pneumococcal meningitis caused by the 6 additional PCV13 types decreased by 66.7%, but the number of cases due to nonvaccine serotypes remained stable. In 2012, 67.6% of cases were caused by the non-PCV13 serotypes including 12F (15%), 24F (15%), 22F (7%), and 15B/C (7%).

## 3. Pneumonia

A marked reduction of incidence of community acquired pneumonia (CAP) mainly in vaccinated children <2 years after the implementation of PCV7 has been reported in a number of countries [19, 20]. However, a number of worldwide studies have reported a significant increase of the incidence of severe CAP cases (i.e., empyema) caused by PCV13 serotypes 1, 3, 5, 7F, and 19A during the large scale administration of PCV7 [21]. Therefore, it was hypothesized that PCV13 could reduce the incidence of CAP in both vaccinated children and unvaccinated subjects.

This hypothesis was confirmed with the implementation of PCV13 in a number of countries. In Uruguay, PCV7 (schedule 2 + 1) was applied in 2008 and replaced by PCV13 (schedule 2 + 1) in 2010, and a catch-up immunization with a single dose of PCV13 was offered to children born between 2005 and 2009. It has been shown that PCV7 and the PCV13 schedules have significantly reduced the incidence of CAP in children 0–14 years of age compared to the CAP incidence prior to the vaccination in 2003–2007 and the introduction of PCV13 had further reduced CAP compared to the CAP incidence during the PCV7 administration period [22]. Particularly, the global CAP hospitalization rate was reduced by 78.1% by PCV7 compared to prior vaccination period and increased to 92.4% after PCV13. In addition, PCV13 introduction was accompanied by a relevant reduction of the incidence of CAP due to serotypes 1 and 5, which, on the contrary, increased in the years of PCV7 administration. However, a significant increase of the total number of CAPs caused by non-PCV13 serotypes was observed during the period of 2009–2012 [22].

A PCV13 schedule 3 + 0 (i.e., 3 doses at 2, 4, and 6 months without any booster) was introduced in Nicaragua in 2010. Becker-Dreps et al. compared the rates of pneumonia hospitalizations and ambulatory visits of CAP between the prevaccine (2008–2010) and vaccine (2011-2012) periods [23]. They found that the adjusted incidence ratio for all-cause CAP hospitalization between the vaccine and prevaccine periods was 0.67 (0.59–0.75) and 0.74 (0.67–0.81) among infants and 1-year-old children, respectively. The adjusted incidence ratio for ambulatory visits of CAP was 0.87 (0.75–1.01) and 0.84 (0.74–0.95) among infants and 1-year-old children, respectively. The low rates of health facility visits due to CAP among children of 2–4 years of age and 5–14 years of age were also observed, suggesting a significant herd immunity effect [23].

The pneumococcal serotypes 1, 3, 5, 7F, and 19A were the most frequently causes of CAP after the implementation of PCV7 and the switch from PCV7 to PCV13 in June 2010 in France, where an observational prospective study was conducted in 8 pediatric emergency departments between June 2009 and May 2012 CAP [24]. Children between 1 month and 15 years of age were included in this study and the CAP was confirmed through chest radiography examination. The incidence of CAP was compared among three periods (1 year for each period) including a pre-PCV13 (2009-2010), a transitional (2010-2011), and a post-PCV13 period (2011-2012). A total of 5,645 children with CAP including 365 and 136 cases of pleural effusion and pneumococcal laboratory-confirmed CAP, respectively, were analyzed. While no catch-up program was performed for children aged 2–5 years, all-cause CAP and pneumococcal cases decreased by 16% and 63%, respectively (*P* < 0.001), in the post-PCV13 periods compared to the pre-PCV13 period. Although the highest reduction was seen in vaccinated children, a significant decrease of CAP incidence was also observed in older unvaccinated children, confirming the herd effect induced by pneumococcal conjugate vaccines. In addition, the number of pleural effusion cases decreased by 53% (*P* < 0.001) and the number of CAPs caused by the additional PCV13 serotypes decreased by 74% [24] in the post-PCV13 period.

## 4. Acute Otitis Media (AOM)

The introduction of PCV7 immediately reduced the office visits of AOM by 6%–7.8% and antibiotic prescriptions by 5.7% [25]. PCV7 vaccination had an even more significant impact on recurrent AOM by reducing tympanostomy tube placements by 20%–24% [26]. From the point of view of microbiologists, PCV7 initially induced a significant reduction of the incidence of pneumococcal AOM caused by the PCV7 serotypes. However, the incidence of AOM, caused by non-PCV7 serotypes, particularly serotypes 6A, 6C, and 19A, increased with the reduction of AOM cases caused by PCV7 serotypes [27, 28]. In addition, PCV13 could significantly reduce the nasopharyngeal colonization by emerging serotypes causing AOM. Therefore, it has been concluded that PCV13 provided better protective effects against AOM than PCV7 [29]. This conclusion was confirmed by the prospective study conducted in Southern Israel by Ben-Shimol et al. In this study, the effects of PCV7/PCV13 sequential introduction on pneumococcal and overall AOM necessitating middle ear fluid (MEF) culture in children <2 years of age were evaluated based on 6,122 AOM cases including 1,893 pneumococcal cases [30]. Compared to the prevaccination period, the incidence of AOM caused by serotypes PCV7 + 6A and the 5 additional PCV13 serotypes 1, 3, 5, 7F, and 19A decreased by 96% and 85%, respectively (IRR and 95% CI = 0.04, 0.02–0.08 and 0.15, 0.07–0.30, resp.). Specifically, in the PCV7 vaccination period, only the incidence of AOM caused by PCV7 + 6A serotypes decreased and in the PCV13 vaccination period the incidence of AOM caused by serotypes 1, 3, 5, 7F, and 19A decreased along with a further PCV7 + 6A AOM reduction. A nonsignificant increase of AOPM caused by non-PCV13 serotypes was observed (IRR = 1.07; 95% CI, 0.72–1.58). In total, the incidence of all-pneumococcal and all-cause AOM decreased by 77% and 60% reductions, respectively [30].

Zhao et al. have evaluated the microbiological characteristics of middle ear effusions of 118 pediatric patients (6 months to 12 years of age) undergoing pressure equalization tube (PET) placement between August 2012 and April 2013 [31]. A total of 39 middle ear cultures from 29 patients led to the growth of at least one bacterial pathogen. Among these patients, 7 only received PCV7, 18 only received PCV13, and 4 received a combination of PCV7 and PCV13. Only one culture from a child who has received PCV7 was positive of* S. pneumoniae* serotype 16. On the contrary,* Haemophilus influenzae* and* Moraxella catarrhalis* were isolated from 7 and 3 cases, respectively. The limited number of* S. pneumoniae* strains isolated from MEF in this study suggests that PCV13 was effective for preventing AOM caused by serotypes covered by the vaccine. One of the limitations of this study was that the studied population has previously received antibiotics treatment repeatedly; therefore, the bacterial strains isolated from the patients were not fully associated with the effectiveness of vaccination of PCV7 and PCV13.

## 5. Nasopharyngeal Carriage

Nasopharyngeal pneumococcal carriage is considered as a prerequisite for the development of pneumococcal disease; therefore, reduction of nasopharyngeal carriage through PCV7 is also useful for reducing the incidence of pneumococcal infections among vaccinated children, their families, and the community [32]. In addition, monitoring the changes of nasopharyngeal carriage induced by PCV13 vaccination is important for the evaluation of vaccine effectiveness and monitoring the development of replacement allows us to predict possible emergence of new serotypes causing pneumococcal diseases.

Cohen et al. have analyzed 943 nasopharyngeal swabs from children (6 to 24 months of age) with AOM between October 2010 and March 2011. Among the 943 children with AOM, 651 received at least 1 dose of PCV13 and 285 received only PCV7 [18]. The overall pneumococcal carriage and carriage of non-PCV7 serotypes in the PCV13-vaccinated children were significantly lower than those in children exclusively vaccinated with PCV7 (53.9% versus 64.6%, *P* = 0.002, and 9.5% versus 20.7%, *P* < 0.0001, resp.). For serotypes 19A, 7F, and 6C, the pneumococcal carriage rates were also significantly lower in PCV13-vaccinated patients than in patients vaccinated only with PCV7 (7.5% versus 15.4%, *P* < 0.001, 0.5% versus 2.8%, *P* = 0.002, and 3.7% versus 8.4%, *P* = 0.003, resp.). Analyses of the other new serotypes included in PCV13 were not available due to the low number of cases identified in the study.

A study on the surveillance of pneumococcal carriage in children <60 months was conducted in July 2010 at a pediatric center in Boston, USA [33]. A total of 1,050* S. pneumoniae* strains were isolated from 1,042 children. Eighty-nine isolates (8.5%) were identified as one of the 6 additional serotypes included in PCV13. A fall/winter peak of pneumococcal carriage of PCV13 serotypes was observed in nonvaccinated children but was blunted in vaccinated children. The authors reported a 74% reduction of PCV13 serotype colonization in the vaccinated children compared to nonvaccinated children. Approximately >75% community children received PCV13 vaccination, and a >50% decline of PCV13 serotype carriage was observed in nonimmune children in the same community. Consequently, the differences of the PCV13 serotype colonization between nonvaccinated and vaccinated children became not significant [33]. In addition, no evidence of replacement has been observed to date.

In England, the pneumococcal carriage of a group of children and their families in 2012 and 2013 after the PCV13 implementation was studied and compared with that in two previous periods, 2001-2002 before the PCV7 introduction and 2008-2009 after the PCV7 introduction [34]. The prevalence of pneumococcal carriage in children <5 years was similar among these three periods, 47.7% (95% CI, 41.8–53.5), 51.0% (95% CI, 44.0–58.0), and 48.4% (95% CI, 44.1–52.7) in 2012-2013, 2008-2009, and 2001/2002, respectively. The prevalence of pneumococcal carriage in children of 5–20-years of age was 22.3% (95% CI, 15.6–30.9) in 2012-2013 and most strains (22/25, 88.0%) were of non-PCV13 serotypes. Only 3.4% (95% CI, 1.9–6.1) children ≥20 years of age were positive of pneumococcal carriage in the period of 2012-2-013, which was lower than that of the last two periods. Compared to the pneumococcal carriage in 2001-2002 before the PCV7 introduction, the odds of PCV7 serotype carriage significantly decreased in both 2008-2009 and 2012/2013, while the odds of carriage of the additional six PCV13 serotypes increased after the PCV7 introduction but significantly declined after the PCV13 introduction. The case/carrier ratio (CCR) for the serotypes of the highest carriage was relatively low. The highest CCR was observed for serotypes 7F, 19A, 3, 8, and 33F. Across the three carriage studies, CCR estimates were stable for nearly all serotypes.

## 6. Conclusions

Despite the difficulties deriving from the differences in immunization programs, vaccination coverage, the timing of PCV13 introduction since previous PCV7 implementation, and the presence/absence of catch-up campaigns, global evaluation and comparison of the incidence of pneumococcal diseases in young children who received PCV13 and/or PCV7 suggest that PCV13 provides a wider and more optimal coverage against pneumococcal disease than PCV7. It has been reported that PCV13 vaccination resulted in significantly higher effectiveness against IPD, mucosal pneumococcal diseases, and pneumococcal carriage than PCV7. Given the high safety and tolerance, PCV13 is an effective conjugate vaccine for controlling the incidence of pneumococcal diseases. However, PCV13 has been implemented for a relatively short time and long-term surveillance should be conducted in the future to further evaluate the safety and effectiveness of this novel vaccine. First of all, effectiveness against the additional 6 serotypes covered by PCV13 should be better defined. If the effectiveness against serotypes 6A, 7F, and 19A is indisputable, the effectiveness against serotypes 1, 3, and 5 needs further evaluation. Due to the relatively low number of cases caused by serotypes 1 and 5, no conclusion was drawn on the effectiveness against these two serotypes between pre- and post-PCV13 implementation periods. However, the reduction of the incidence of CAP with empyema, a condition frequently caused by the most invasive serotypes, suggests that PCV13 was highly effective in preventing these pathogens [24]. It has been reported that the effectiveness of PCV13 against serotype 3 was not satisfactory [14]. Finally, the replacement phenomenon should be further studied due to inconsistent results.
